# Prognostic performance of thymidine kinase 1 activity in patients with hormone receptor-positive and HER2-negative metastatic breast cancer treated with CDK4/6 and aromatase inhibitors

**DOI:** 10.1007/s10549-025-07879-0

**Published:** 2026-02-11

**Authors:** Nicole L. Brown, Sacha J. Howell, Dimitrios Papantoniou, Olle Eriksson, Mattias Bergqvist, Amy Williams, Amy Kavanagh, Alexandra Backlund, Ahmed Albu-Kareem, Ellinor Elinder, Karolina Larsson, Monika Uminska, Maria Ekholm

**Affiliations:** 1 The Christie NHS Foundation Trust, Manchester, M20 4BX UK; 2 Division of Cancer Sciences, Faculty of Biology, Medicine and Health, The University of Manchester, Manchester, M13 9PL UK; 3 Ryhov County Hospital, Department of Oncology, SE-551 85 Jönköping, Sweden; 4 Futurum – The Academy for Health and Care, SE-551 85 Jönköping, Region Jönköping County Sweden; 5 Biovica International AB, SE-752 37 Uppsala, Sweden; 6 Linköping University Hospital, Department of Oncology, SE-581 85 Linköping, Sweden; 7 Södersjukhuset, Department of Oncology, SE-118 83 Stockholm, Sweden; 8 Department of Oncology, Sahlgrenska University Hospital, SE-413 45 Gothenburg, Sweden; 9 Department of Oncology, Institute of Clinical Sciences, Sahlgrenska Academy, University of Gothenburg, SE-405 30 Gothenburg, Sweden; 10 Kalmar County Hospital, Department of Oncology, SE-391 85 Kalmar, Sweden; 11 Division of Surgery, Orthopedics, and Oncology, Department of Biomedical and Clinical Sciences, Linköping University, SE-581 83 Linköping, Sweden

**Keywords:** Advanced breast cancer, CDK4/6 inhibitors, Aromatase inhibitors, Prognostic biomarker, Thymidine kinase 1, Survival analysis

## Abstract

**Purpose:**

Thymidine kinase 1 activity (TKa) has previously been demonstrated as a prognostic biomarker for progression-free survival (PFS) in hormone receptor-positive, HER2-negative metastatic breast cancer (MBC), but its optimal use remains undefined. This study evaluated the prognostic performance of TKa across multiple sampling time points and thresholds in patients receiving first-line CDK4/6 inhibitor plus aromatase inhibitor therapy.

**Methods:**

TKa was measured (DiviTum® assay) at baseline (BL), cycle 1 day 15 (C1D15), and cycle 2 day 1 (C2D1) in patients enrolled in the PDM-MBC study (*n* = 90). Thresholds for PFS discrimination were identified using maximally selected rank statistics, with predefined cut-offs tested for comparison. Prognostic performance was assessed using the corrected Akaike information criterion (AICc) and Harrell’s concordance index (C-index).

**Results:**

TKa was prognostic at all time points. Data-derived thresholds identified groups with differing PFS and overall survival (OS), and predefined cut-offs (≥ 50, ≥ 100, ≥ 250 DiviTum® units of Activity [DuA]) also discriminated survival outcomes. BL and C2D1 models performed better than C1D15 and comparably to multi-time-point models. Among patients with BL TKa ≥ 250 DuA, suppression to < 100 DuA at C1D15 was associated with longer median PFS (23.9 vs. 10.3 months; *p* = 0.034).

**Conclusion:**

Baseline TKa provides prognostic information, with potential added value from repeated testing in those with high baseline levels. TKa behaves as a continuous biomarker of risk, and continuous modelling may offer more clinically informative individual risk estimation, while thresholds may retain value for specific clinical contexts. Validation in larger cohorts is warranted to support integration into routine practice.

**Supplementary Information:**

The online version contains supplementary material available at 10.1007/s10549-025-07879-0.

## Introduction

Cyclin dependent kinase 4/6 inhibitors (CDK4/6i) have improved outcomes for patients with hormone receptor-positive (HR+) and human epidermal growth factor receptor-negative (HER2−) metastatic breast cancer (MBC), with median progression-free survival (mPFS) on first-line CDK4/6i plus aromatase inhibitor (AI) therapy exceeding two years [1–3]. Regular imaging is required to evaluate treatment response, leading to unnecessary imaging procedures for patients with long-term response to therapy. These procedures can cause physical and psychological discomfort [4] and place burden on healthcare systems. Conversely, patients with endocrine-resistant tumours may benefit from earlier switching to alternative therapies, though identifying such patients prospectively remains challenging. There is a clinical need for a more personalised approach to disease monitoring.

Thymidine kinase 1 (TK1) is an enzyme involved in DNA synthesis and cell proliferation [5], with elevated levels typically observed in patients with cancer compared to healthy individuals [6]. TK1 activity (TKa) has demonstrated prognostic value in HR+/HER2− MBC, with higher baseline TKa associated with worse survival outcomes, and decreasing TKa levels typically observed following initiation of endocrine-based therapy [7–14]. Paoletti et al. showed that higher TKa levels, both at baseline and when measured repeatedly after treatment initiation, were independently associated with shorter progression-free survival (PFS) and overall survival (OS) in the SWOG S2206 trial of first-line AI ± fulvestrant [11]. Malorni et al. demonstrated similar prognostic value of TKa in the BioItaLEE study, including patients treated with first-line CDK4/6i plus AI [14]. CDK4/6is induce G1 cell cycle arrest, resulting in decreased TKa, with rebound levels following drug washout correlating with recovery of tumour proliferation [15, 16]. Malorni et al. evaluated the combined prognostic value of cycle 1 day 15 (C1D15) and cycle 2 day 1 (C2D1) (TKa dynamics), and showed that, among patients with suppressed levels at C1D15, continued suppression versus rebound at C2D1 further discriminated PFS outcomes [14]. Despite growing evidence supporting TKa as a prognostic biomarker in ER+/HER2− MBC, its optimal clinical use, particularly in relation to cut-off values and sampling time points, remains to be defined.

In this analysis from the prospective Personalised Disease Monitoring in Metastatic Breast Cancer (PDM-MBC) study, we aimed to evaluate the optimal use of TKa as a prognostic biomarker for survival outcomes in patients treated with CDK4/6i plus AI therapy. Specifically, we assessed the prognostic performance of models including TKa as a continuous variable and dichotomised at exploratory thresholds across multiple sampling time points.

## Methods

### Study design

Detailed information about the PDM-MBC study (Clinical Trial Registration: NCT04597580) has been published previously [17]. In brief, 97 patients with HR+/HER2− MBC eligible for first-line CDK4/6i (ribociclib or palbociclib) plus an AI, with the addition of a gonadotropin-releasing hormone analogue in premenopausal women, were enrolled across six sites in the United Kingdom and Sweden. CDK4/6i were typically administered on days 1–21 of a 28-day cycle, followed by a 7-day break (days 22–28). Blood samples were collected at baseline (BL), C1D15, and C2D1, and thereafter every 3–4 months, both during treatment (‘on’) and after the treatment break (‘off’) in the CDK4/6i dosing cycle, until progressive disease (PD) or study discontinuation for other reasons. Imaging was performed every 3–4 months and included computed tomography of the thorax, abdomen, and pelvis, with or without magnetic resonance imaging. PD was defined as radiological progression per RECIST 1.1 criteria [18], or clinical progression as determined by the treating oncologist. Follow-up is ongoing until May 2026.

### TKa sampling and analysis

Blood samples were drawn into a single 10 mL BD Vacutainer serum tube (Becton Dickinson), allowed to clot at room temperature for ~ 30 min, and then centrifuged at 2000 g for 10 min. Serum was aliquoted into 1.0 mL tubes and stored at − 70 to − 80 °C. TKa was retrospectively analysed using the FDA approved DiviTum® TKa assay (Biovica International, Uppsala, Sweden) [19], and results were not shared with treating oncologists. The assay measures bromodeoxyuridine incorporation into DNA strands immobilised on the reaction plate, followed by ELISA-based colourimetric quantification using an alkaline phosphatase-conjugated anti-BrdU antibody [15, 20].

### Statistical analysis

Although precise quantification below the lower limit of quantification (< 50 DiviTum® units of Activity [DuA]) is not commercially reported per manufacturer specifications [20], the full range of TKa values was retained to preserve potentially important information. Wilcoxon signed-rank tests were used to compare changes in TKa levels between time points, with Bonferroni correction applied for multiple comparisons. Samples were included in prognostic analyses if patients completed cycle 1 without interruption. C1D15 samples were included if collected 12–18 days ‘on’ CDK4/6i treatment, and C2D1 samples were included if collected 5–10 days ‘off’ CDK4/6i treatment. PFS and OS were defined as the time from treatment initiation to the respective event. Patients without events were censored at the date of last imaging (PFS) or last known alive date (OS). TKa was analysed as a continuous variable (ln-transformed), a dichotomised variable, and as a three-level variable reflecting TKa dynamics at C1D15 and C2D1: Low-Low, Low–High, and High regardless of C2D1 level (High-Any). The maximally selected rank statistics method [21] was used to define optimal thresholds for PFS discrimination, and additional predefined cut-offs were tested for comparison with previous literature [14]. In addition, absolute change (Δ ln TKa) and fold-change (ln TKa ratio) between time points were evaluated. Kaplan–Meier analysis and log-rank tests were used to compare survival outcomes. Cox proportional hazards models were used to estimate hazard ratios (HRs) for TKa and survival outcomes, with multivariable adjustment for age, disease-free interval, and visceral disease. Model performance was assessed using the corrected Akaike information criterion (AICc) [22], which adjusts for small sample size. Generally, lower AIC values indicate better model fit; for models fitted to the same dataset, ΔAIC ≤ 2 suggests similar performance, while ΔAIC > 4 indicates preference for the model with the lower AIC [23]. Model discriminative ability was estimated with the optimism-corrected (from 1000 bootstrap resamples) Harrell’s concordance index (C-index) [24]. Receiver operating characteristic (ROC) curve analysis was used to evaluate the ability of TKa to identify patients with PFS < 6 months. Area under the curve (AUC), sensitivity, specificity, positive predictive value (PPV), and negative predictive value (NPV) are reported for each time point. *P*-values < 0.05 were considered statistically significant, except where multiple comparisons were corrected using the Bonferroni method (adjusted significance threshold *α* = 0.017). All statistical analyses were performed using R version 4.5.0 (R Core Team, 2025). Figures were created in Microsoft PowerPoint, with final formatting performed in Adobe Illustrator (Adobe inc., USA). This study is reported in accordance with the REMARK guidelines [25].

## Results

### Patient cohort and survival outcomes

**Fig. 1:**
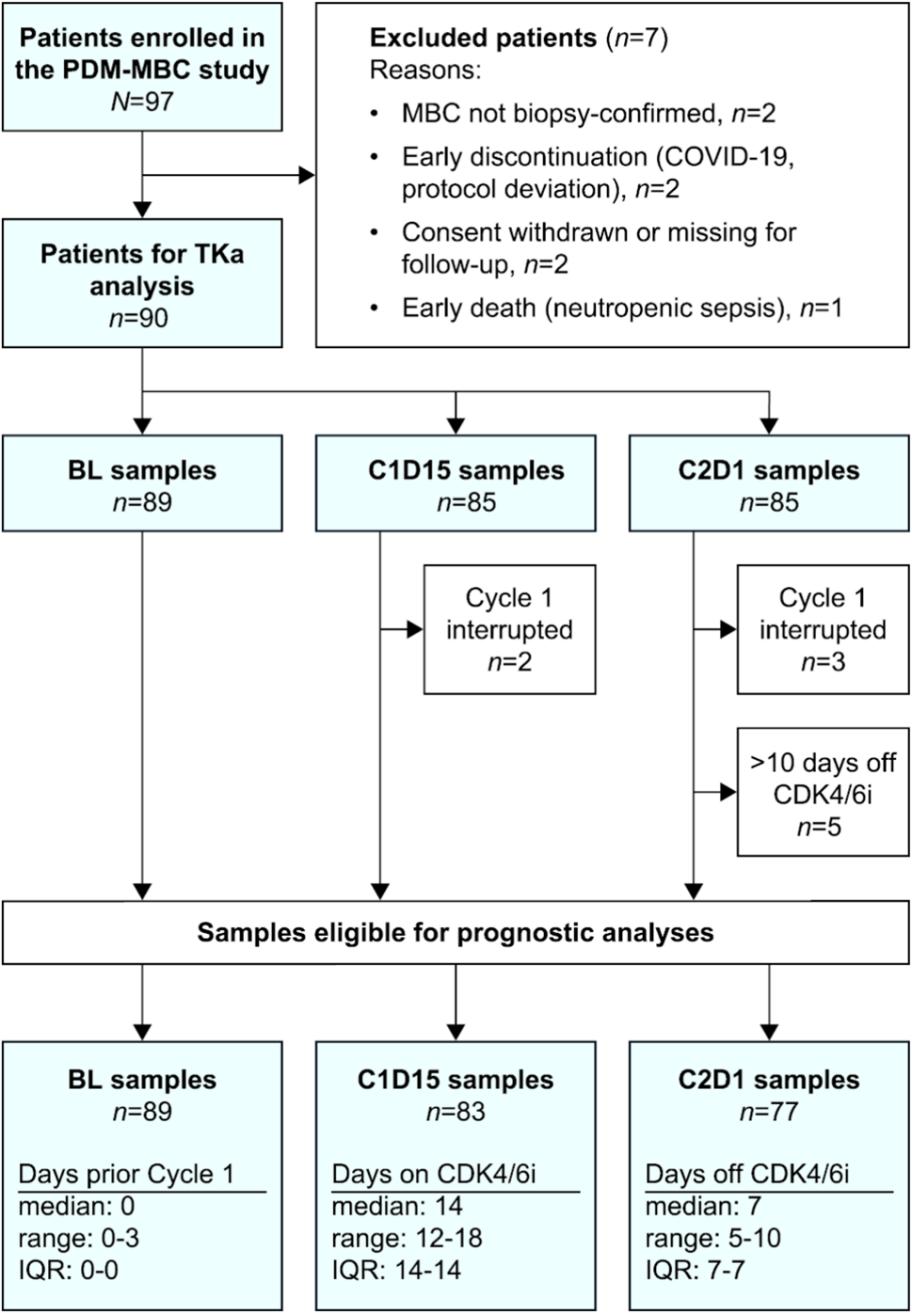
CONSORT diagram. Abbreviations: BL, baseline; C1D15, cycle 1 day 15; C2D1, cycle 2 day 1; IQR, interquartile range; MBC, metastatic breast cancer; TKa, thymidine kinase 1 activity

Ninety patients were eligible for TKa analysis, of whom 89, 83, and 77 patients met the criteria for prognostic analyses at BL, C1D15, and C2D1, respectively. Reasons for exclusion are shown in Fig. 1, and patient and disease characteristics are provided in Table 1.

**Table 1:** Patient demographics and disease characteristics

Variable	All patients (*n* = 90)
Age, years, median (IQR)	61 (51–70)
Sex, *n* (%)	
Women	89 (98.9)
Men	1 (1.1)
Menopausal status, *n* (%)	
Premenopausal	21 (23.3)
Perimenopausal	1 (1.1)
Postmenopausal	67 (74.4)
Not applicable (male)	1 (1.1)
Performance status, *n* (%)	
0	57 (63.3)
1	28 (31.1)
2	5 (5.6)
Disease setting, *n* (%)	
*De novo* metastatic	32 (35.6)
Recurrent metastatic	58 (64.5)
Disease-free interval^a^	
Years, median (IQR)	12.0 (6.5–16.6)
< 5 years, *n* (%)	8 (13.8)
≥ 5 years,* n* (%)	50 (86.2)
Prior adjuvant systemic therapy^a^, *n* (%)	
Chemotherapy	
Yes	22 (37.9)
No	35 (60.3)
Unknown	1 (1.7)
Endocrine therapy	
Yes	49 (84.5)
No	7 (12.1)
Unknown	2 (3.4)
Bisphosphonates	
Yes	3 (5.2)
No	55 (94.8)
Presence of visceral metastases, *n* (%)	59 (65.6)
Progression-free survival	
Months, median (95% CI)	23.6 (16.0–NE)
Events, *n* (%)	50 (55.6)
Overall survival	
Months, median (95% CI)	59.8 (38.3–NE)
Events, *n* (%)	31 (34.4)

^a^For patients with recurrent disease (*n* = 58)

Abbreviations: CI, confidence interval; IQR, interquartile range; *n*, number of patients; NE, not estimable

At data cut-off, 50 patients (55.6%) had developed PD, and 31 (34.4%) had died. mPFS and median OS (mOS) were 23.6 months (95% confidence interval [CI],16.0–not estimable [NE]) and 59.8 months (95% CI, 38.3–NE), respectively. Median follow-up time for patients without events was 29.9 months for PFS (range, 0.5–68.6) and 32.5 months for OS (range, 23.1–68.6).

### TKa levels at baseline and early time points

**Fig. 2:**
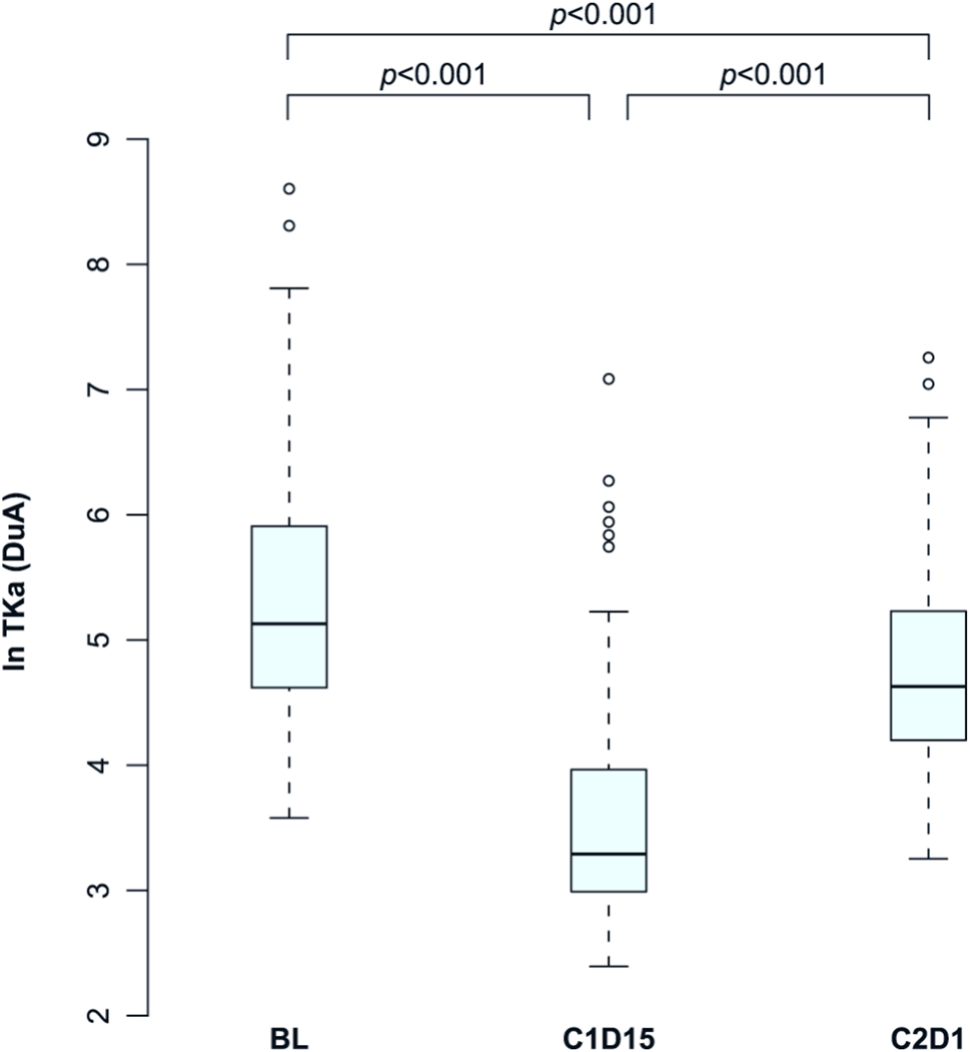
Median ln-transformed TKa levels at baseline, cycle 1 day 15, and cycle 2 day 1 in all patients (*n* = 90). Abbreviations: BL, baseline; C1D15, cycle 1 day 15; C2D1, cycle 2 day 1; DuA. DiviTum® units of Activity; TKa, thymidine kinase 1 activity

Median TKa was 170 DuA (range, 36–5480) at BL, 27 DuA (range, 11–1201) at C1D15 and 103 DuA (range, 26–1424) at C2D1. TKa significantly declined from BL to C1D15 (*p* < 0.001), followed by a rebound at C2D1 (*p* < 0.001), though remaining below BL levels (*p* < 0.001). ln-transformed TKa levels with interquartile ranges are shown in Fig. 2.

### Comparison of TKa-based models for predicting PFS

Optimal thresholds to discriminate PFS in this cohort were < 160 DuA at BL, < 71 DuA at C1D15, and < 87 DuA at C2D1. The BL and C2D1 single time-point models (ln TKa or optimal cut-offs) outperformed C1D15, with ΔAICc > 4 and higher C-index values (Table 2). Models based on absolute change or fold-change between time points performed worse (Online Resource 1 [Supplementary Table 1]). For TKa dynamics, an exploratory threshold of < 100 DuA performed better than < 145 DuA, employed in BioItaLEE [14], which was based on the assay’s previous lower limit of quantification (Table 2). Neither the TKa dynamics model nor models combining ln TKa at two (BL + C1D15, BL + C2D1, C1D15 + C2D1) or three time points (BL + TKa Dynamics), with or without interaction terms, outperformed the BL or C2D1 models alone. However, an interaction was observed between ln TKa at BL and TKa dynamics (< 145 DuA) (*p* = 0.035), with a similar trend for TKa dynamics (< 100 DuA) (*p* = 0.061) (Table 2). Full AICc and C-index values for additional thresholds (≥ 50, ≥ 100, and ≥ 250 DuA) are provided in Online Resource 1 (Supplementary Table 1).

**Table 2:** Prognostic performance of TKa models for progression-free survival based on single-time-point, TKa dynamics, and multi-time-point models in patients with evaluable TKa data at all three time points (*n* = 74)

Model type	AICc	C-index^a^	*p*-value
Single-time-point models			
Continuous variable (ln TKa)			
BL C1D15 C2D1	305.9 311.2 304.2	0.670 0.641 0.691	– – –
Two groups (Low vs. High TKa)			
BL (Low TKa: < 160 DuA^b^)	305.5	0.649	–
C1D15 (Low TKa: < 71 DuA^b^)	310.2	0.635	–
C2D1 TKa (Low TKa: < 87 DuA^b^)	304.8	0.651	–

TKa dynamics models			
TKa dynamics^c^			
TKa dynamics (< 100 DuA^c^)	305.2	0.686	–
TKa dynamics (< 145 DuA^c^)	309.1	0.657	–

Multi-time-point models			
Continuous variables (ln TKa)			
BL + C1D15	307.2	0.655	–
BL + C1D15 + interaction	307.8	0.656	*p* = 0.20
BL + C2D1	306.2	0.677	–
BL + C2D1 + interaction	304.7	0.680	*p* = 0.066
C1D15 + C2D1	306.0	0.682	–
C1D15 + C2D1 + interaction	306.8	0.674	*p* = 0.21
Continuous variable (TKa dynamics + ln TKa)			
TKa dynamics (< 100 DuA^c^) + BL	306.8	0.682	–
TKa dynamics (< 100 DuA^c^) + BL + interaction	305.6	0.673	*p* = 0.061
TKa dynamics (< 145 DuA^c^) + BL	308.2	0.663	–
TKa dynamics (< 145 DuA^c^) + BL + interaction	306.2	0.664	*p* = 0.036

^a^Optimism-corrected Harrell’s C-index from 1000 bootstrap resamples

^b^Threshold for optimal PFS discrimination was determined by the maximally selected rank statistics method

^c^Categorised by TKa levels at C1D15 and C2D1 using predefined cut-off values (Low-Low; Low-High, or High-Any)

Abbreviations: BL, baseline; C1D15, cycle 1 day 15; C2D1, cycle 2 day 15; DuA, DiviTum® units of Activity; TKa, thymidine kinase 1 activity

### Impact of TKa cut-offs on PFS and OS discrimination

**Fig. 3:**
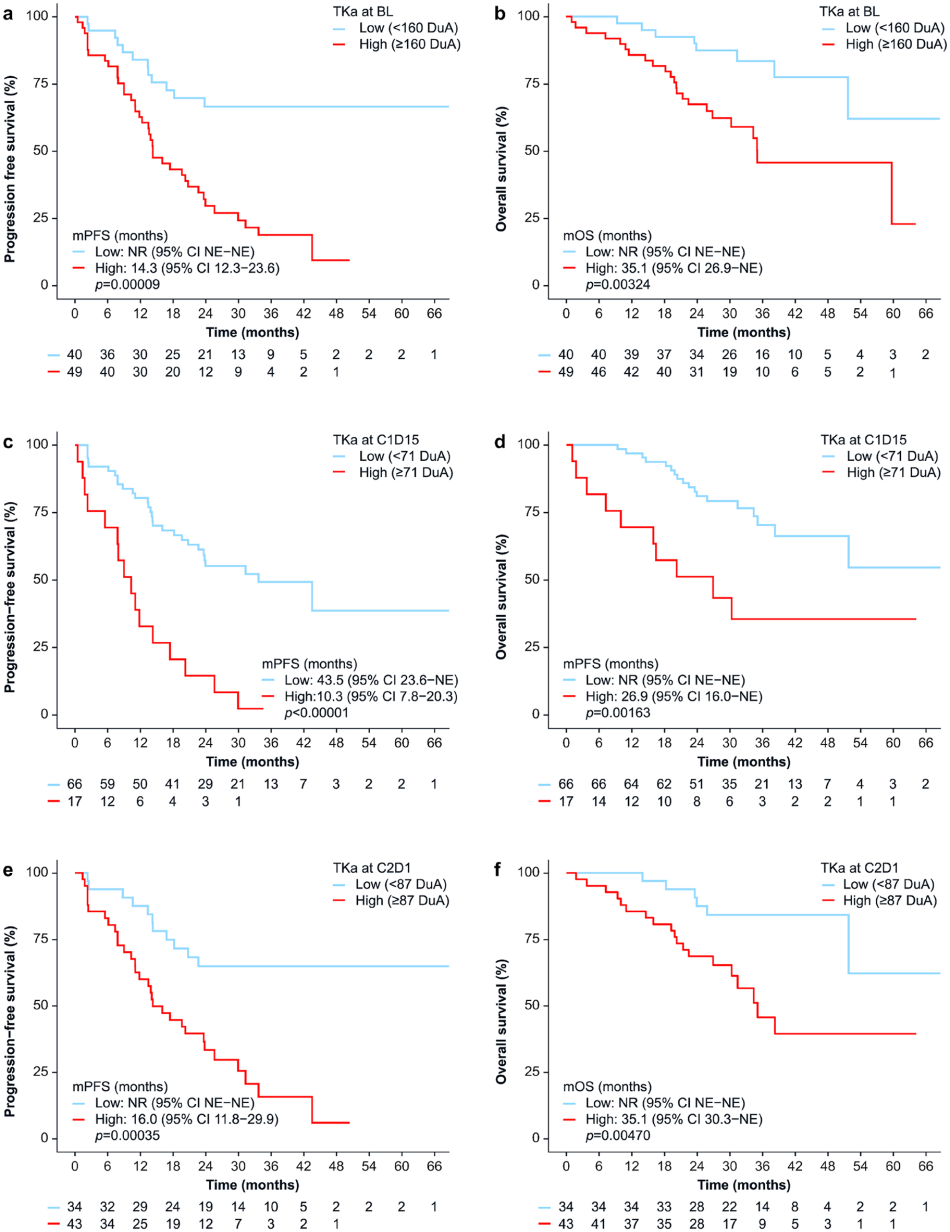

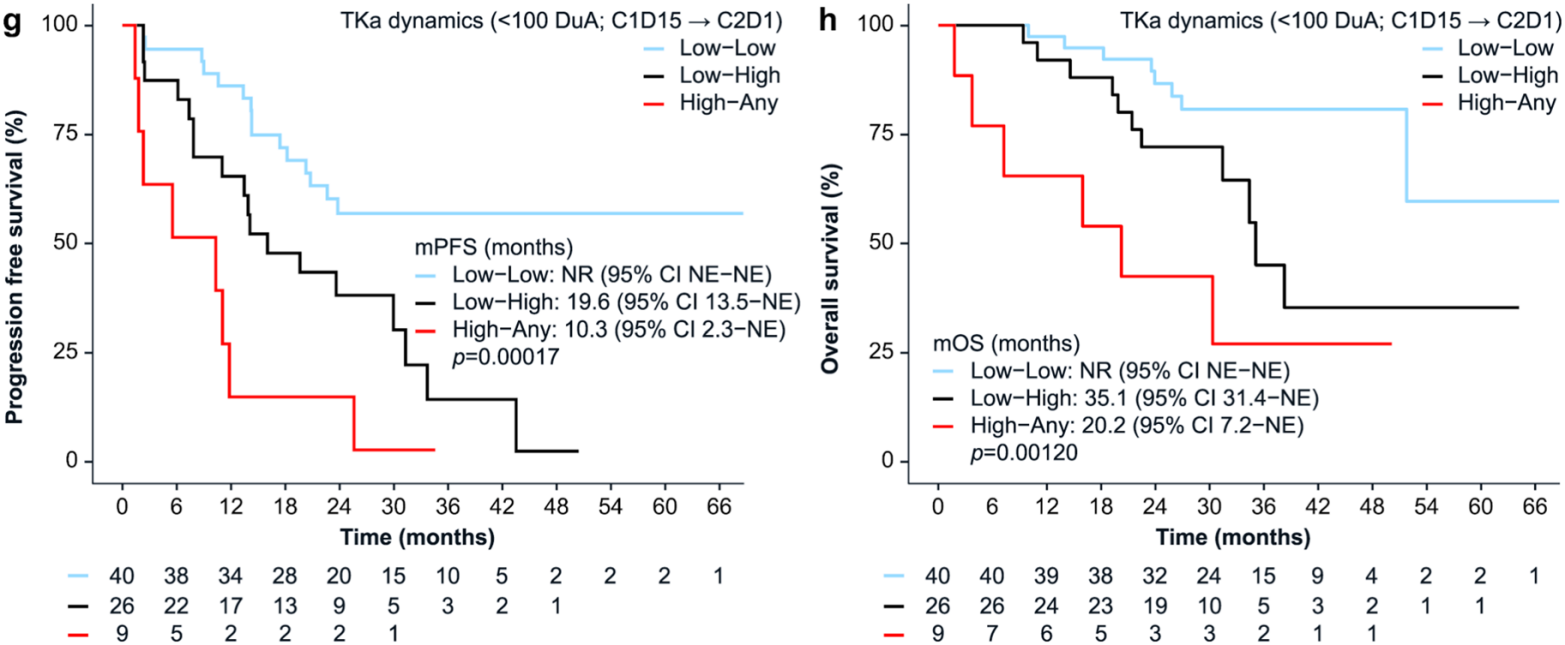
Kaplan–Meier curves of progression-free survival (PFS) and overall survival (OS) stratified by TKa levels at baseline (panels a and b), at cycle 1 day 15 (panels c and d), at cycle 2 day 1 (panels e and f), and for TKa dynamics (panels g and h). For TKa dynamics, low TKa at C1D15 and C2D1 was defined as < 100 DuA. Median PFS and OS are shown with 95% CIs and log-rank *p*-values. Numbers below the curves indicate the number of patients at risk in each group. Abbreviations: BL, baseline; C1D15, cycle 1 day 15; C2D1, cycle 2 day 1; CI, confidence interval; DuA, DiviTum® units of Activity; NE, not estimable; NR, not reached; TKa, thymidine kinase 1 activity

Patients with low TKa levels (based on optimal cut-offs) had significantly longer PFS and OS at all time points (Fig. 3a–f, Table 3). TKa remained significantly associated with both PFS and OS after adjustment for age, disease-free interval, and visceral metastases (Table 3). Pre-defined thresholds (≥ 50, ≥ 100, and ≥ 250 DuA) also significantly discriminated PFS and OS in most cases. Full results from Kaplan-Meier and Cox regression analyses across all time points and cut-off values are presented in Online Resource 2 (Supplementary Tables 2a–c). When patients were grouped by TKa dynamics using the < 100 DuA cut-off, significant differences in PFS and OS were observed across the three groups. The longest PFS was seen in those with suppressed levels at both time points (Low-Low), and the shortest in those with TKa ≥ 100 DuA at C1D15, regardless of level at C2D1 (High-Any) (Fig. 3g–h, Table 3). Results for TKa dynamics using the ≥ 145 DuA cut-off are provided in Online Resource 2 (Supplementary Tables 2d). Among patients with BL TKa ≥ 250 DuA (n = 32), those whose TKa was suppressed to < 100 DuA at C1D15 (n = 21) had a longer mPFS than those with TKa ≥ 100 DuA (23.9 versus 10.3 months; HR = 2.48; 95% CI, 1.07–5.72; *p* = 0.034).

**Table 3:** Progression-free survival and overall survival stratified by TKa (Low vs. High) at baseline, cycle 1 day 15, and cycle 2 day, and by TKa dynamics

Progression-free survival							
Group, *n* (%)	Events *n* (%)	mPFS months (95% CI)	*p*-value^b^	HR (95% CI)	*p*-value^c^	Adjusted HR (95% CI)	*p*-value^d^
BL TKa (< 160 DuA^a^)							
Low, 40 (45)	12 (30.0)	NR (NE-NE)	< 0.001	Ref		Ref	
High, 49 (55)	38 (77.6)	14.3 (12.3–23.6)		3.40 (1.77–6.52)	< 0.001	3.02 (1.53–5.94)	0.001
C1D15 TKa (< 71 DuA^a^)							
Low, 66 (80)	29 (43.9)	43.5 (23.6–NE)	< 0.001	Ref		Ref	
High, 17 (20)	16 (94.1)	10.3 (7.8–20.3)		3.86 (2.07–7.21)	< 0.001	3.45 (1.65–7.22)	0.001
C2D1 TKa (< 87 DuA^a^)							
Low, 34 (44)	11 (32.4)	NR (NE–NE)	< 0.001	Ref		Ref	
High, 43 (56)	31 (72.1)	16.0 (11.8–29.9)		3.30 (1.65–6.60)	< 0.001	2.88 (1.40–5.92)	0.004
TKa dynamics^e^ (< 100 DuA)							
Low-Low, 40 (53)	15 (37.5)	NR (23.8–NE)	< 0.001	Ref		Ref	
Low–High, 26 (35)	18 (69.2)	19.6 (13.5–NE)		2.41 (1.21–4.79)	0.012	2.41 (1.21–4.83)	0.013
High-Any, 9 (12)	8 (88.9)	10.3 (2.3–NE)		5.26 (2.21–12.5)	< 0.001	4.03 (1.58–10.3)	0.004

Overall survival							
Group, *n* (%)	Events *n* (%)	mOS months (95% CI)	*p*-value^b^	HR (95% CI)	*p*-value^c^	Adjusted HR (95% CI)	*p*-value^d^
BL TKa (< 160 DuA^a^)							
Low, 40 (45)	8 (20.0)	NR (51.8–NE)	0.003	Ref		Ref	
High, 49 (55)	23 (46.9)	35.1 (26.9–NE)		3.17 (1.41–7.11)	0.005	3.35 (1.45–7.74)	0.005
C1D15 TKa (< 71 DuA^a^)							
Low, 66 (80)	18 (27.3)	NR (51.8–NE)	0.002	Ref		Ref	
High, 17 (20)	10 (58.8)	26.9 (16.0–NE)		3.25 (1.50–7.07)	0.003	3.78 (1.62–8.79)	0.002
C2D1 TKa (< 87 DuA^a^)							
Low, 34 (44)	6 (17.6)	NR (51.8–NE)	0.005	Ref		Ref	
High, 43 (56)	19 (44.1)	35.1 (30.3–NE)		3.54 (1.39–9.00)	0.008	3.39 (1.31–8.75)	0.012
TKa dynamics^e^ (< 100 DuA)							
Low-Low, 40 (53)	8 (20.0)	NR (51.8–NE)		Ref		Ref	
Low–High, 26 (35)	11 (44.2)	35.1 (31.4–NE)	0.001	2.61 (1.04–6.52)	0.041	2.52 (1.00–6.38)	0.051
High-Any, 9 (12)	6 (66.7)	20.2 (7.2–NE)		6.19 (2.12–18.11)	< 0.001	6.51 (2.14–19.8)	0.001

^a^Threshold for optimal PFS discrimination was determined by the maximally selected rank statistics method

^b^Log-rank test

^c^Univariable Cox proportional hazards analysis

^d^Multivariable Cox proportional hazards analyses, adjusted for age, disease-free interval, and presence of visceral metastases

^e^Categorised by TKa levels at C1D15 and C2D1 using predefined cut-off values

Abbreviations: BL, baseline; C1D15, cycle 1 day 15; C2D1, cycle 2 day 1; CI, confidence interval; DuA, DiviTum® units of Activity; HR, hazard ratio; mOS, median overall survival; mPFS, median progression-free survival; NE, not estimable; NR, not reached; Ref, reference; TKa, thymidine kinase 1 activity

### Performance of early TKa assessment for predicting early progression

We evaluated whether TKa could identify patients at risk of early progression (within 6 months). Optimal cut-offs were ≥ 589 DuA at BL, ≥ 35.5 DuA at C1D15, and ≥ 193 DuA at C2D1, and using these, C2D1 showed the strongest performance, with an AUC of 0.815 (95% CI, 0.658–0.971), sensitivity of 77.8%, specificity of 87.9%, PPV of 46.7% and NPV of 96.7%. Data for BL and C1D15 are shown in Table 4**.**

**Table 4:** Performance of TKa for predicting early progression within 6 months

		Patients (*n*)	AUC	95% CI	*p*-value	Sensitivity (%)	Specificity (%)	PPV (%)	NPV (%)
BL TKa (≥ 589 DuA^a^)		86	0.736	0.549–0.922	0.014	60.0	85.5	35.3	94.2
C1D15 TKa (≥ 50 DuA^b^)		81	0.778	0.620–0.937	0.001	60.0	76.7	26.1	93.1
C2D1 TKa (≥ 193 DuA^a^)		75	0.815	0.658–0.971	< 0.001	77.8	87.9	46.7	96.7

^a^Threshold for progression within 6 months was determined by the maximally selected rank statistics method.

^b^A threshold of ≥ 35.5 DuA was identified, but since this value was below the lower limit of quantification, ≥ 50 DuA was used for calculations.

Abbreviations: AUC, area under the curve; BL, baseline; CI, confidence interval; C1D15, cycle 1 day 15; C2D1, cycle 2 day 1; DuA, DiviTum® units of Activity; NPV, negative predictive value, PPV, positive predictive value; TKa, thymidine kinase 1 activity

## Discussion

In this analysis from the PDM-MBC study, we show that TKa was prognostic for both PFS and OS in patients receiving first-line CDK4/6i plus AI for HR + /HER2 − MBC. These results are in line with findings from several other MBC cohorts treated with diverse systemic therapies across first- and later-lines settings [7–14, 26, 27]. Our study adds to this evidence by systematically assessing the prognostic value of multiple early TKa measurements and by evaluating both data-derived and predefined thresholds within the current first-line setting.

The median TKa at BL, C1D15, and C2D1 was 174 DuA, < 145 DuA (previous lower limit of detection), and 159 DuA in the BioItaLEE study, compared to 174 DuA, 27 DuA, and 103 DuA in our cohort. High TKa has typically been defined as values above the median in previous studies [7–14, 26]. The thresholds that most effectively discriminated PFS in our study were < 160 DuA at BL, < 71 DuA at C1D15, and < 87 DuA at C2D1, all of which were also significantly associated with OS. Given that these data-driven thresholds may have limited clinical applicability, we also explored alternative fixed cut-offs (< 50 DuA, < 100 DuA, and < 250 DuA). These cut-offs retained prognostic discrimination in most comparisons, although some analyses were underpowered due to small subgroup sizes. These findings indicate that the prognostic information provided by TKa is not defined by a specific threshold. Instead, TKa reflects a continuous risk, with higher levels corresponding to progressively shorter survival outcomes. Importantly, thresholds are not inherently meaningful unless linked to a clinical decision.

The BL and C2D1 single-time-point models provide comparable prognostic information for PFS, but sampling at baseline may be more reliable from a clinical perspective, as it is unaffected by treatment interruptions or sampling delays. C2D1 testing, however, may serve as an alternative when baseline samples are unavailable. The inferior prognostic performance of C1D15 models likely reflects the high proportion of samples with TKa suppressed to < 50 DuA (> 70%), where assay precision is reduced. Neither the TKa dynamics model nor other multi-time-point models showed superior prognostic performance to BL or C2D1 alone. Interestingly, heterogeneity was observed among patients with baseline TKa ≥ 250 DuA, with longer PFS in those whose TKa was suppressed to < 100 DuA at C1D15. Although these findings should be interpreted as prognostic, they raise interest in evaluating TKa as a predictive marker for the addition of CDK4/6i in prospective randomised trials, especially as subgroup analyses based on clinicopathological characteristics have not identified any population that achieves comparable outcomes on endocrine therapy alone [1–3]. An interaction between ln TKa at BL and TKa dynamics was also noted, although not statistically significant at lower thresholds, and this also requires validation. To conclude, while repeated TKa testing during the first treatment cycle did not improve overall model performance, our findings suggest that certain subgroups of patients may still benefit from repeated TKa testing to obtain more refined estimates of PFS.

We also explored whether TKa could help identify patients at risk of early progression, with the strongest prognostic association observed at C2D1. The modest positive predictive value likely reflects the low event rate in our cohort. Further investigation in larger cohorts is needed to determine whether early TKa measurements can reliably identify rapid progressors and to define clinically meaningful thresholds for this specific purpose.

The PDM-MBC study was designed as an exploratory monitoring study. While the sample size was adequate for hypothesis-generating analyses of early prognostic biomarkers, it was not powered for definitive validation. Strengths include its prospective design, a patient cohort representative of routine clinical practice, and the systematic evaluation of TKa across multiple early time points and thresholds. In addition, the strict inclusion criteria for prognostic analyses, applied to minimise variability associated with sampling timing, treatment interruptions, or intercurrent illness, enhance the reliability of the TKa results. Nonetheless, the modest sample size and some missing samples remain important limitations.

## Conclusion

TKa showed consistent associations with survival outcomes across the tested time points and thresholds in this HR+/HER2− MBC cohort treated with first-line CDK4/6i plus AI therapy. Baseline testing is practical from a clinical standpoint, and although repeated measurements may provide added value in selected subgroups, multi-time-point models were overall not superior to BL or C2D1 assessments alone. In the future, a more clinically useful estimation of individual patient risk may be achieved by incorporating TKa as a continuous biomarker in prognostic models rather than applying fixed thresholds. As individual risk estimation may facilitate personalised monitoring strategies, further validation of TKa as a prognostic, and potentially also predictive, biomarker in larger cohorts and in prospective randomised trials is warranted.

## Supplementary Information

Below is the link to the electronic supplementary material.

Supplementary file1 (DOC 100 KB)

Supplementary file2 (DOC 233 KB)

## Data Availability

The datasets generated and/or analysed during the current study are not publicly available due to patient privacy and confidentiality restrictions but are available from the corresponding author on reasonable request.

## Abbreviations

AI: Aromatase inhibitor
AICc: Corrected Akaike information criterion
BL: Baseline
CDK4/6i: Cyclin dependent kinase 4/6 inhibitor
C1D15: Cycle 1 Day 15
C2D1: Cycle 2 Day 1
DuA: DiviTum® units of Activity
HER2 −: Human epidermal growth factor receptor 2 negative
HR: Hazard ratio
HR +: Hormone receptor-positive
MBC: Metastatic breast cancer
mOS: Median overall survival
mPFS: Median progression-free survival
OS: Overall survival
PD: Progressive disease
PFS: Progression-free survival
RECIST: Response evaluation criteria in solid tumours
REMARK: Reporting recommendations for tumour marker prognostic studies
TK1: Thymidine kinase 1
TKa: Thymidine kinase 1 activity
