# Supplementary material for: Prognostic performance of thymidine kinase 1 activity in patients with hormone receptor-positive and HER2-negative metastatic breast cancer treated with CDK4/6 and aromatase inhibitors
Source: Breast Cancer Res Treat. 2026 Feb 11;216(1):6. doi: 10.1007/s10549-025-07879-0 (PMC12894156; doi:10.1007/s10549-025-07879-0)
Supplement: Supplementary file 1 — Supplementary file1 (DOC 100 KB) [file 10549_2025_7879_MOESM1_ESM.doc]

**Supplementary Table 1**

**Prognostic performance of thymidine kinase 1 activity in patients with hormone receptor-positive and HER2-negative metastatic breast cancer treated with CDK4/6 and aromatase inhibitors**

Nicole L Brown, Sacha J Howell, Dimitrios Papantoniou, Olle Eriksson, Mattias Bergqvist, Amy Williams, Amy Kavanagh, Alexandra Backlund, Ahmed Albu-Kareem, Ellinor Elinder, Karolina Larsson, Monika Uminska, and Maria Ekholm.

*Breast Cancer Res Treat*

**Supplementary Table 1.** Prognostic performance of TKa models for progression-free survival based on predefined cut-off values, absolute changes (Δ), and fold changes (ratios) between time points in patients with evaluable TKa data at all three time points (n=74).

| **Model type** | **AICc** | **C-index^a^** |
| --- | --- | --- |
| Single time-point models |  |  |
| TKa at BL (Low vs. High) |  |  |
| Cut-off: <50 DuA | 309.6 | 0.635 |
| Cut-off: <100 DuA | 312.1 | 0.607 |
| Cut-off: <250 DuA | 311.0 | 0.634 |
| TKa at C1D15 (Low vs. High) |  |  |
| Cut-off: <50 DuA | 313.7 | 0.624 |
| Cut-off: <100 DuA | 311.4 | 0.645 |
| Cut-off: <250 DuA | 315.2 | 0.612 |
| TKa at C2D1 (Low vs. High) |  |  |
| Cut-off: <50 DuA | 302.0 | 0.656 |
| Cut-off: <100 DuA | 306.1 | 0.666 |
| Cut-off: <250 DuA | 310.8 | 0.661 |
| Multi time-point models |  |  |
| Change between time points, ln TKa (Δ) |  |  |
| BL - C1D15 | 310.8 | 0.611 |
| BL - C2D1 | 315.4 | 0.598 |
| C1D15 - C2D1 | 311.9 | 0.611 |
| Fold change between time points, ln TKa (ratio) |  |  |
| C1D15 / BL | 315.0 | 0.593 |
| C2D1 / BL | 315.1 | 0.602 |
| C2D1 / C1D15 | 314.0 | 0.591 |
| 1. optimism-corrected Harrell’s C-index from 1000 bootstrap resamples.   Abbreviations: AICc, corrected Akaike information criterion; BL, baseline; C1D15, cycle 1 day 15; C2D1, cycle 2 day 1; C-index, Harrell’s concordance index; DuA, DiviTum units of activity; PFS, progression-free survival; TKa, thymidine kinase 1 activity. | | |
