# Supplementary material for: Prognostic performance of thymidine kinase 1 activity in patients with hormone receptor-positive and HER2-negative metastatic breast cancer treated with CDK4/6 and aromatase inhibitors
Source: Breast Cancer Res Treat. 2026 Feb 11;216(1):6. doi: 10.1007/s10549-025-07879-0 (PMC12894156; doi:10.1007/s10549-025-07879-0)
Supplement: Supplementary file 2 — Supplementary file2 (DOC 233 KB) [file 10549_2025_7879_MOESM2_ESM.doc]

**Supplementary Tables 2 a-d**

**Prognostic performance of thymidine kinase 1 activity in patients with hormone receptor-positive and HER2-negative metastatic breast cancer treated with CDK4/6 and aromatase inhibitors**

Nicole L Brown, Sacha J Howell, Dimitrios Papantoniou, Olle Eriksson, Mattias Bergqvist, Amy Williams, Amy Kavanagh, Alexandra Backlund, Ahmed Albu-Kareem, Ellinor Elinder, Karolina Larsson, Monika Uminska, and Maria Ekholm.

*Breast Cancer Res Treat*

**Supplementary Table 2a.** Progression-free survival and overall survival stratified by TKa (Low vs. High) at baseline using optimal and predefined cut-off values.

| **Progression-free survival** | | | | | | | |
| --- | --- | --- | --- | --- | --- | --- | --- |
| **TKa at baseline, *n* (%)** | **Events, *n* (%)** | **mPFS, months (95% CI)** | ***p*-value^b^** | **HR (95% CI)** | ***p*-value^c^** | **Adjusted HR (95% CI)** | ***p*-value^d^** |
| Cut-off: <50 DuA |  |  |  |  |  |  |  |
| Low, 4 (5) | 0 (0) | NR (NE-NE) | 0.049 | Ref. |  | Ref. |  |
| High, 85 (96) | 50 (58.8) | 20.8 (14.3 – 33.7) |  | NE (NE – NE) | NE | NE (NE – NE) | NE |
| Cut-off: <100 DuA |  |  |  |  |  |  |  |
| Low, 19 (21) | 5 (26.3) | NR (16.8 – NE) | 0.020 | Ref. |  | Ref. |  |
| High, 70 (79) | 45 (64.3) | 19.6 (13.9 – 31.3) |  | 2.85 (1.13 – 7.19) | 0.026 | 2.49 (0.96 – 6.45) | 0.061 |
| Cut-off: <160 DuA^a^ |  |  |  |  |  |  |  |
| Low, 40 (45) | 12 (30.0) | NR (NE – NE) | <0.001 | Ref. |  | Ref. |  |
| High, 49 (55) | 38 (77.6) | 14.3 (12.3 – 23.6) |  | 3.40 (1.77 – 6.52) | <0.001 | 3.02 (1.53 – 5.94) | 0.001 |
| Cut-off: <250 DuA |  |  |  |  |  |  |  |
| Low, 55 (62) | 25 (45.5) | 43.5 (18.2 – NE) | 0.007 | Ref. |  | Ref. |  |
| High, 34 (38) | 25 (73.5) | 13.5 (9.0 – 29.9) |  | 2.13 (1.22 – 3.71) | 0.008 | 1.83 (1.02 – 3.26) | 0.041 |
| **Overall survival** | | | | | | | |
| **TKa at baseline, *n* (%)** | **Events, *n* (%)** | **mOS, months (95% CI)** | ***p-*value^b^** | **HR (95% CI)** | ***p*-value^c^** | **Adjusted HR (95% CI)** | ***p-*value^d^** |
| Cut-off: <50 DuA |  |  |  |  |  |  |  |
| Low, 4 (5) | 0 (0) | NR (NE– NE) | 0.17 | Ref. |  | Ref. |  |
| High, 85 (96) | 31 (36.5) | 59.8 (38.3 – NE) |  | NE (NE – NE) | NE | NE (NE – NE) | NE |
| Cut-off: <100 DuA |  |  |  |  |  |  |  |
| Low, 19 (21) | 4 (14.8) | NR (NE– NE) | 0.11 | Ref. |  | Ref. |  |
| High, 70 (79) | 27 (38.6) | 51.8 (35.1 – NE) |  | 2.28 (0.80 – 6.54) | 0.12 | 2.66 (0.88 – 8.03) | 0.082 |
| Cut-off: <160 DuA^a^ |  |  |  |  |  |  |  |
| Low, 40 (45) | 8 (20.0) | NR (51.8 – NE) | 0.032 | Ref. |  | Ref. |  |
| High, 49 (55) | 23 (46.9) | 35.1 (26.9 – NE) |  | 3.17 (1.41 – 7.11) | 0.005 | 3.35 (1.45 – 7.74) | 0.005 |
| Cut-off: <250 DuA |  |  |  |  |  |  |  |
| Low, 55 (62) | 12 (21.8) | NR (51.8 – NE) | <0.001 | Ref. |  | Ref. |  |
| High, 34 (38) | 19 (55.9) | 34.4 (21.4 – NE) |  | 3.29 (1.59 – 6.78) | 0.001 | 3.25 (1.56 – 6.81) | 0.002 |
| ^a^ threshold for optimal PFS discrimination was determined by the maximally selected rank statistics method, ^b^ log-rank test, ^c^ univariable Cox proportional hazards analysis, ^d^ multivariable Cox proportional hazards analyses, adjusted for age, disease-free interval, and presence of visceral metastases.  Abbreviations: BL, baseline; CI, confidence interval; DuA, DiviTum units of activity; HR, hazard ratio; mOS, median overall survival; mPFS, median progression-free survival; NE, not estimable; NR, not reached; Ref, reference; TKa, thymidine kinase 1 activity. | | | | | | | |

**Supplementary Table 2b.** Progression-free survival and overall survival stratified by TKa (Low vs. High) at cycle 1 day 15 using optimal and predefined cut-off values.

| **Progression-free survival** | | | | | | | |
| --- | --- | --- | --- | --- | --- | --- | --- |
| **TKa at C1D15, *n* (%)** | **Events, *n* (%)** | **mPFS, months (95% CI)** | ***p*-value^b^** | **HR (95% CI)** | ***p*-value^c^** | **Adjusted HR (95% CI)** | ***p*-value^d^** |
| Cut-off: <50 DuA |  |  |  |  |  |  |  |
| Low, 59 (71) | 27 (45.8) | 43.5 (23.6 – NE) | 0.002 | Ref. |  | Ref. |  |
| High, 24 (29) | 18 (75.0) | 11.8 (7.9 – 29.9) |  | 2.51 (1.38 – 4.59) | 0.003 | 2.05 (1.02 – 4.12) | 0.044 |
| Cut-off: <71 DuA^a^ |  |  |  |  |  |  |  |
| Low, 66 (80) | 29 (43.9) | 43.5 (23.6 – NE) | <0.001 | Ref. |  | Ref. |  |
| High, 17 (20) | 16 (94.1) | 10.3 (7.8 – 20.3) |  | 3.86 (2.07 – 7.21) | <0.001 | 3.45 (1.65 – 7.22) | 0.001 |
| Cut-off: <100 DuA |  |  |  |  |  |  |  |
| Low, 71 (86) | 34 (47.9) | 31.3 (22.6 – NE) | <0.001 | Ref. |  | Ref. |  |
| High, 12 (14) | 11 (91.6) | 9.1 (2.3 – NE) |  | 3.95 (1.98 – 7.90) | <0.001 | 3.16 (1.45 – 6.90) | 0.004 |
| Cut-off: <250 DuA |  |  |  |  |  |  |  |
| Low, 77 (93) | 40 (51.9) | 23.9 (19.6 – NE) | 0.045 | Ref. |  | Ref. |  |
| High, 6 (7) | 5 (83.3) | 8.7 (1.8 – NE) |  | 2.49 (0.98 – 6.33) | 0.055 | 1.70 (0.59 – 4.85) | 0.32 |
| **Overall survival** | | | | | | | |
| **TKa at C1D15, *n* (%)** | **Events, *n* (%)** | **mOS, months (95% CI)** | ***p-*value^b^** | **HR (95% CI)** | ***p*-value^c^** | **Adjusted HR (95% CI)** | ***p-*value^d^** |
| Cut-off: <50 DuA |  |  |  |  |  |  |  |
| Low, 59 (71) | 16 (27.1) | NR (51.8 – NE) | 0.018 | Ref. |  | Ref. |  |
| High, 24 (29) | 12 (50.0) | 30.3 (20.3 – NE) |  | 2.41 (1.14 – 5.11) | 0.021 | 2.49 (1.09 – 5.68) | 0.031 |
| Cut-off: <71 DuA^a^ |  |  |  |  |  |  |  |
| Low, 66 (80) | 18 (27.3) | NR (51.8 – NE) | 0.002 | Ref. |  | Ref. |  |
| High, 17 (20) | 10 (58.8) | 26.9 (16.0– NE) |  | 3.25 (1.50 – 7.07) | 0.003 | 3.78 (1.62 – 8.79) | 0.002 |
| Cut-off: <100 DuA |  |  |  |  |  |  |  |
| Low, 71 (86) | 20 (28.2) | NR (51.8 – NE) | <0.001 | Ref. |  | Ref. |  |
| High, 12 (14) | 8 (66.7) | 23.6 (7.2 – NE) |  | 3.90 (1.70 – 8.94) | 0.001 | 4.07 (1.70 – 9.72) | 0.002 |
| Cut-off: <250 DuA |  |  |  |  |  |  |  |
| Low, 77 (93) | 24 (31.1) | NR (51.8 – NE) | 0.019 | Ref. |  | Ref. |  |
| High, 6 (7) | 4 (66.7) | 18.8 (1.8 – NE) |  | 3.32 (1.154– 9.64) | 0.027 | 3.72 (1.15 – 12.0) | 0.028 |
| ^a^ threshold for optimal PFS discrimination was determined by the maximally selected rank statistics method, ^b^ log-rank test, ^c^ univariable Cox proportional hazards analysis, ^d^ multivariable Cox proportional hazards analyses, adjusted for age, disease-free interval, and presence of visceral metastases.  Abbreviations: C1D15, cycle 1 day 15; CI, confidence interval; DuA, DiviTum units of activity; HR, hazard ratio; mOS, median overall survival; mPFS, median progression-free survival; NE, not estimable; NR, not reached; Ref, reference; TKa, thymidine kinase 1 activity. | | | | | | | |

**Supplementary Table 2c.** Progression-free survival and overall survival stratified by TKa (Low vs. High) at cycle 2 day 1 using optimal and predefined cut-off values.

| **Progression-free survival** | | | | | | | |
| --- | --- | --- | --- | --- | --- | --- | --- |
| **TKa at C2D1, *n* (%)** | **Events, *n* (%)** | **mPFS, months (95% CI)** | ***p*-value^b^** | **HR (95% CI)** | ***p*-value^c^** | **Adjusted HR (95% CI)** | ***p*-value^d^** |
| Cut-off: <50 DuA |  |  |  |  |  |  |  |
| Low, 9 (12) | 0 (0) | NR (NE – NE) | 0.004 | Ref. |  | Ref. |  |
| High, 68 (88) | 42 (61.8) | 20.8 (14.3 – 33.7) |  | NE (NE – NE) | NE | NE (NE – NE) | NE |
| Cut-off: <87 DuA^a^ |  |  |  |  |  |  |  |
| Low, 34 (44) | 11 (32.4) | NR (NE – NE) | <0.001 | Ref. |  | Ref. |  |
| High, 43 (56) | 31 (72.1) | 16.0 (11.8 – 29.9) |  | 3.30 (1.65 – 6.60) | <0.001 | 2.88 (1.40 – 5.92) | 0.004 |
| Cut-off: <100 DuA |  |  |  |  |  |  |  |
| Low, 41 (53) | 16 (39.0) | NR (22.6 – NE) | 0.001 | Ref. |  | Ref. |  |
| High, 36 (47) | 26 (72.2) | 14.1 (11.0 – 31.3) |  | 2.75 (1.47 – 5.14) | 0.002 | 2.58 (1.37 – 4.86) | 0.003 |
| Cut-off: <250 DuA |  |  |  |  |  |  |  |
| Low, 66 (88) | 33 (50.0) | 31.3 (20.3 – NE) | 0.002 | Ref. |  | Ref. |  |
| High, 11 (12) | 9 (81.8) | 7.8 (2.3 – NE) |  | 3.13 (1.48 – 6.58) | 0.003 | 2.61 (1.18 – 5.76) | 0.017 |
| **Overall survival** | | | | | | | |
| **TKa at C2D1, *n* (%)** | **Events, *n* (%)** | **mOS, months (95% CI)** | ***p*-value^b^** | **HR (95% CI)** | ***p*-value^c^** | **Adjusted HR (95% CI)** | ***p*-value^d^** |
| Cut-off: <50 DuA |  |  |  |  |  |  |  |
| Low, 9 (12) | 0 (0) | NR (NE – NE) | 0.034 | Ref. |  | Ref. |  |
| High, 68 (88) | 25 (36.8) | 51.8 (35.1 – NE) |  | NE (NE – NE) | NE | NE (NE – NE) | NE |
| Cut-off: <87 DuA^a^ |  |  |  |  |  |  |  |
| Low, 34 (44) | 6 (17.6) | NR (51.8 – NE) | 0.005 | Ref. |  | Ref. |  |
| High, 43 (56) | 19 (44.2) | 35.1 (30.3– NE) |  | 3.54 (1.39 – 8.97) | 0.008 | 3.39 (1.31 – 8.75) | 0.012 |
| Cut-off: <100 DuA |  |  |  |  |  |  |  |
| Low, 41 (53) | 8 (19.5) | NR (51.8 – NE) | 0.005 | Ref. |  | Ref. |  |
| High, 36 (47) | 17 (47.2) | 35.1 (30.3 – NE) |  | 3.19 (1.37 – 7.44) | 0.007 | 3.12 (1.32 – 7.37) | 0.009 |
| Cut-off: <250 DuA |  |  |  |  |  |  |  |
| Low, 66 (88) | 17 (25.8) | NR (51.8 – NE) | <0.001 | Ref. |  | Ref. |  |
| High, 11 (12) | 8 (72.7) | 20.2 (11.0 – NE) |  | 4.46 (1.90 – 10.5) | <0.001 | 4.47 (1.84 – 10.8) | <0.001 |
| ^a^ threshold for optimal PFS discrimination was determined by the maximally selected rank statistics method, ^b^ log-rank test, ^c^ univariable Cox proportional hazards analysis, ^d^ multivariable Cox proportional hazards analyses, adjusted for age, disease-free interval, and presence of visceral metastases.  Abbreviations: C2D1, cycle 2 day 1; CI, confidence interval; DuA, DiviTum units of activity; HR, hazard ratio; mOS, median overall survival; mPFS, median progression-free survival; NE, not estimable; NR, not reached; Ref, reference; TKa, thymidine kinase 1 activity. | | | | | | | |

**Supplementary Table 2d.** Progression-free survival and overall survival stratified by TKa dynamics using cut-off values of <100 and <145 DuA.

| **Progression-free survival** | | | | | | | |
| --- | --- | --- | --- | --- | --- | --- | --- |
| **TKa dynamics  (C1D15 - C2D1), *n* (%)** | **Events, *n* (%)** | **mPFS, months (95% CI)** | ***p-*value^a^** | **HR (95% CI)** | ***p*-value^b^** | **Adjusted HR (95% CI)** | ***p*-value^c^** |
| Cut-off: <100 DuA |  |  |  |  |  |  |  |
| Low-Low, 40 (53) | 14 (35.9) | NR (23.8 – NE) | <0.001 | Ref. |  | Ref. |  |
| Low-High, 26 (25) | 18 (69.2) | 19.6 (13.5 – NE) |  | 2.41 (1.21 – 4.79) | 0.012 | 2.41 (1.21 – 4.83) | 0.013 |
| High-Any, 9 (12) | 8 (88.9) | 10.3 (2.3 – NE) |  | 5.26 (2.21 – 12.5) | <0.001 | 4.03 (1.58 – 10.3) | 0.004 |
| Cut-off: <145 DuA |  |  |  |  |  |  |  |
| Low-Low, 51 (68) | 23 (45.1) | NR (20.8 – NE) | 0.005 | Ref. |  | Ref. |  |
| Low-High, 17 (23) | 12 (70.6) | 19.6 (10.3 – NE) |  | 2.21 (1.10 – 4.44) | 0.027 | 2.65 (1.28 – 5.49) | 0.009 |
| High-Any, 7 (9) | 6 (85.7) | 11.1 (1.8 – NE) |  | 3.49 (1.41 – 8.63) | 0.007 | 2.51 (0.90 – 6.98) | 0.079 |
| **Overall survival** | | | | | | | |
| **TKa dynamics  (C1D15 - C2D1), *n* (%)** | **Events, *n* (%)** | **mOS, months (95% CI)** | ***p-*value^a^** | **HR (95% CI)** | ***p*-value^b^** | **Adjusted HR (95% CI)** | ***p-*value^c^** |
| Cut-off: <100 DuA |  |  |  |  |  |  |  |
| Low-Low, 40 (53) | 7 (17.9) | NR (51.8 – NE) | 0.001 | Ref. |  | Ref. |  |
| Low-High, 26 (25) | 11 (44.2) | 35.1 (31.4 – NE) |  | 2.61 (1.04 – 6.52) | 0.041 | 2.52 (1.00 – 6.38) | 0.051 |
| High-Any, 9 (12) | 6 (66.7) | 20.2 (7.2 – NE) |  | 6.19 (2.12 – 18.1) | <0.001 | 6.51 (2.14 - 19.8) | 0.001 |
| Cut-off: <145 DuA |  |  |  |  |  |  |  |
| Low-Low, 51 (68) | 13 (25.5) | NR (51.8 – NE) | 0.023 | Ref. |  | Ref. |  |
| Low-High, 17 (23) | 8 (47.1) | 34.4 (22.5 – NE) |  | 2.42 (0.99 – 5.95) | 0.054 | 2.34 (0.94 – 5.81) | 0.068 |
| High-Any, 7 (9) | 4 (57.1) | 30.3 (3.7 – NE) |  | 3.73 (1.20 – 11.6) | 0.023 | 4.32 (1.27 - 14.8) | 0.020 |
| ^a^ log-rank test, ^b^ univariable Cox proportional hazards analysis, ^c^ multivariable Cox proportional hazards analyses, adjusted for age, disease-free interval, and presence of visceral metastases.  Abbreviations: C1D15, cycle 1 day 15; C2D1, cycle 2 day 1; CI, confidence interval; DuA, DiviTum units of activity; HR, hazard ratio; mOS, median overall survival; mPFS, median progression-free survival; NE, not estimable; NR, not reached; Ref, reference; TKa, thymidine kinase 1 activity. | | | | | | | |
